# Anterior fiber‐reinforced ribbon composite resin bridge—A case report

**DOI:** 10.1002/ccr3.1745

**Published:** 2018-08-16

**Authors:** Valentina Pankratz, Stefan Zimmer, Ljubiša Marković

**Affiliations:** ^1^ Department of Operative and Preventive Dentistry Dental School Faculty of Health Witten/Herdecke University Witten Germany

**Keywords:** case report, dental general practice, fiber‐reinforced composite bridge, minimal invasive, operative dentistry

## Abstract

The case describes the fabrication of a unilaterally fixed anterior fiber‐reinforced composite bridge in a 14‐year‐old girl. Using this technique, it is possible to temporarily replace a missing anterior tooth until a definitive restoration can be inserted.

## 1 INTRODUCTION

The upper left lateral incisor of a 14‐year‐old girl was lost because of unsuccessful root canal treatment. It was decided to place a fiber‐reinforced composite bridge with unilateral anchorage as an intermediate solution. After 1 year the restoration was still in service, but some revision was needed.

A 14‐year‐old girl introduced herself with a *dens invaginatus* at our dental school. A *dens invaginatus,* also known as *dens in dente* is the consequence of an invagination of enamel epithelium into the pulp space. The reported prevalence varies widely between 0.3% and 10%.^1^ The most frequently affected teeth are lateral incisors. In their radiographical examination of 766 dental students with 1532 maxillary lateral incisors, Gotoh et al^2^ found 148 cases (9.66%) of *dens invaginatus*.

*Dens invaginatus* shows multiple morphological variations in crown and root formation from type 1 with almost no morphological changes to type 3 which can be associated with periapical infection.^1^ This is caused by the fact that the invagination extends through the root and communicates with the periodontal ligament at the apical foramen. In this case, a type 3 invagination was present on the left upper lateral incisor. As an apical periodontitis was present, the tooth required root canal treatment. Due to the complex tooth morphology, this treatment could not be accomplished successfully and the tooth had to be extracted. An orthodontic therapy permitted to keep open the gap between canine and central incisor. Orthodontic treatment had been completed a few months earlier at our dental school.

**Figure 1:**
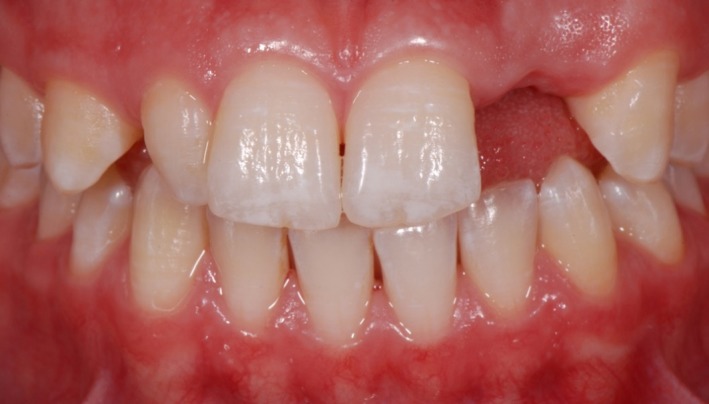
Clinical aspect of the front teeth 5 mo after extraction of the left upper lateral incisor. The right lateral incisor shows the morphology of a conical tooth. For esthetic reasons the conical tooth and the canines had to be included in the esthetical rehabilitation in order to achieve symmetry

The absence of the lateral incisor caused a restriction in phonetics and esthetics which was disturbing the patient (Figure 1). After discussing all treatment options, it was decided to replace the missing tooth by an implant as soon as the girl turned 18 years old. Due to the young age of the patient, immediate implant treatment was not possible. A removable partial denture as temporary solution was not an option for the patient. Therefore, it was decided to place a fiber‐reinforced composite Maryland‐like bridge as an intermediate solution until implantation is possible. No palatal tooth reduction was necessary because the anterior overjet was adequate to place an anchorage.

## 2 CASE PRESENTATION

The lost upper left lateral incisor was replaced by fiber‐reinforced composite bridge with unilateral anchorage which was fixed to the left central incisor. The main problem of the planned restoration was that the space width was too big to get an adequate result by restoring only the missing tooth. Furthermore, attention had to be paid to the hypomineralization of the remaining front teeth. The main concern was to realize an affordable and esthetically appealing rehabilitation of the front teeth in the upper jaw.

As a first step, plaster models were made and the front teeth were remodeled with a wax‐up in the laboratory situation. Afterwards, a silicon mold was prepared in order to establish a mock‐up and use it for an intraoral demonstration for the patient. Furthermore, different colors were chosen and tested intraorally. The nanohybrid composite CeramX^®^ duo (Dentsply Sirona Inc, York, PA, USA) was used as a restorative material. Unbonded composite samples were polymerized on the enamel surface for a more detailed color determination. The patient's occlusion was checked.

**Figure 2:**
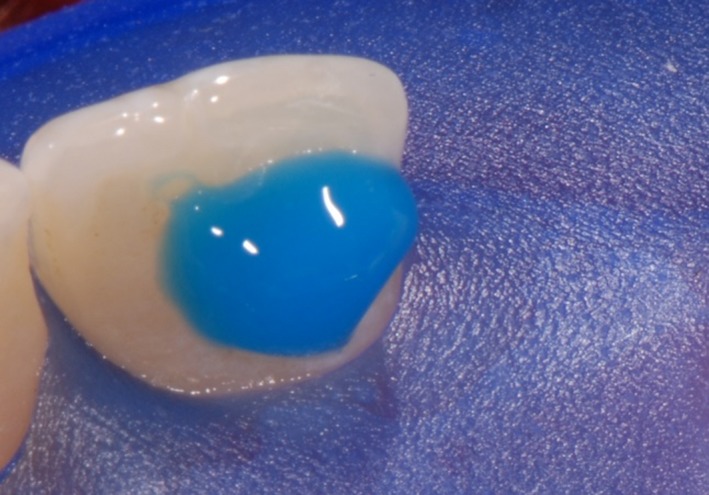
The left middle incisor was etched for 30 s with 36% phosphoric acid and conditioned with XP Bond^®^ (Dentsply Sirona Inc.)

**Figure 3:**
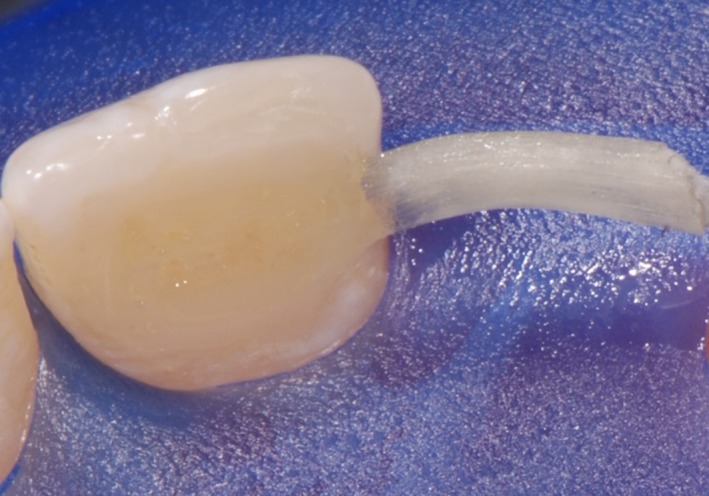
A preimpregnated fiberglass ribbon was fixed with x‐flow^®^ A3 (Dentsply Sirona Inc.). The thickness of the composite between the teeth and ribbon was kept as thin as possible

As a first step, the front teeth were isolated with rubber dam, etched for 30 seconds with 36% phosphoric acid and conditioned with XP Bond^®^ (Dentsply Sirona Inc.; Figure 2). Thereafter, the wax‐up was implemented intraorally using a silicone mold which was applied on the palatal surfaces of the upper front teeth. The mold was used to apply the first layer of composite. A preimpregnated fiberglass ribbon (everStick^®^ C&B; GC EUROPE N.V., Leuven, Belgium) was fixed with x‐flow^®^ A3 (Dentsply Sirona Inc.). The thickness of the composite between teeth and ribbon was kept as thin as possible (Figure 3).

**Figure 4:**
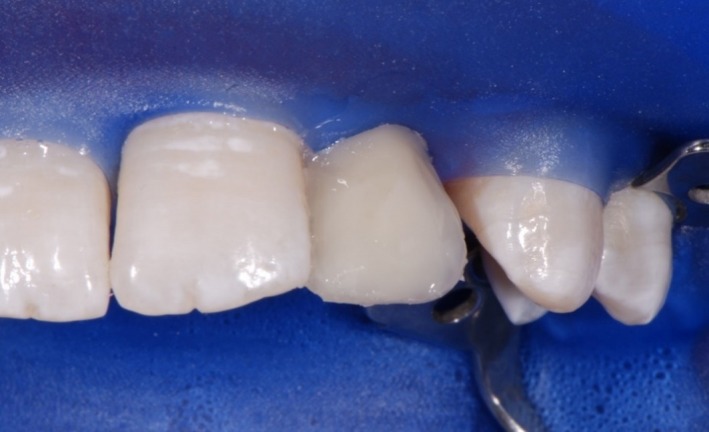
Subsequent dentine core layering with CeramX^®^ duo D1 and D2

**Figure 5:**
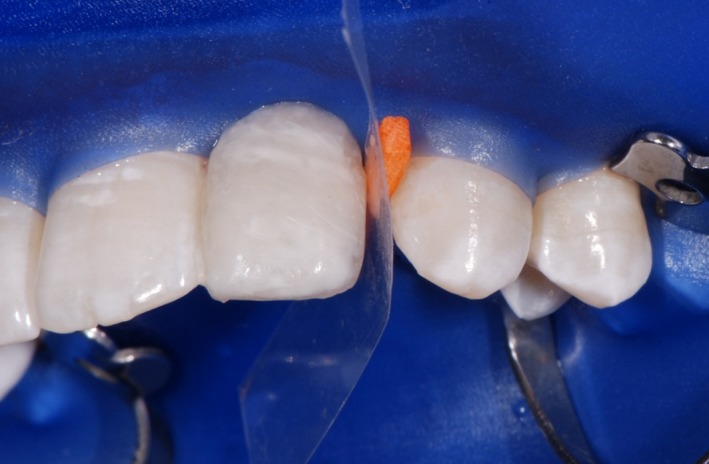
A polyester strip and a wedge were inserted for isolating and modeling the marginal ridges with CeramX^®^ duo E2. Venus^®^ flow baseline (Heraeus Kulzer GmbH) as a snow‐white composite was used to imitate the hypomineralization. Finally, we layered the last enamel imitating composite CeramX^®^ duo E2

The composite was used in combination with the Etch & Rinse adhesive XP Bond^®^ (Dentsply Sirona Inc.). Dentin core layering was performed using the D1 and D2 shades of the composite system (Figure 4). As a next step, a polyester strip and a wedge were inserted for isolating and modeling the marginal ridges with E2 enamel shade (Figure 5). Venus^®^ flow baseline (Heraeus Kulzer GmbH, Hanau, Germany) as a snow‐white composite was used to mimic the hypomineralization which was seen on the upper front teeth. As a final layer, enamel E2 shade was applied.

**Figure 6:**
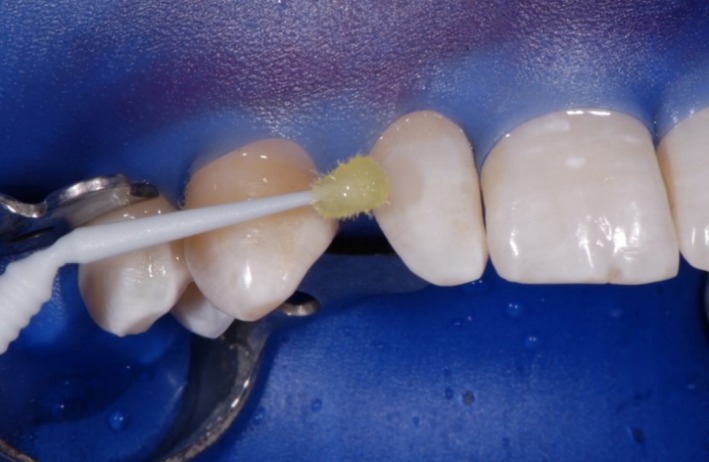
The conically shaped tooth 12 and the canines were prepared as described before

**Figure 7:**
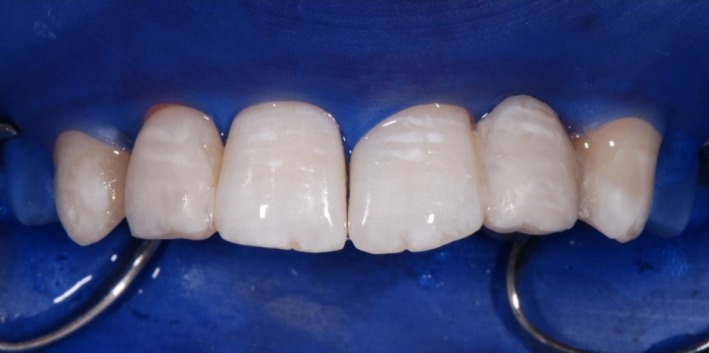
The conically shaped tooth 12 and the canines were enlarged. The figure shows the final result before finishing and polishing

**Figure 8:**
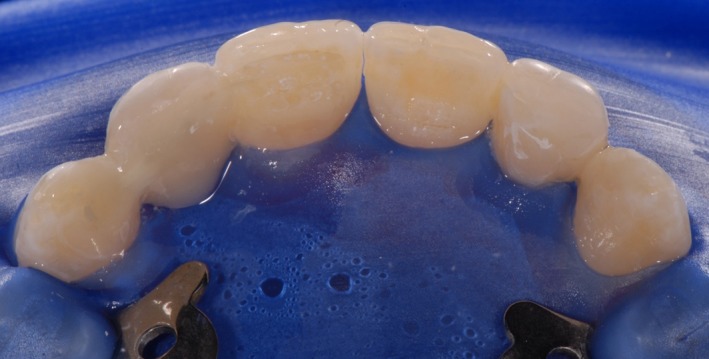
A precise surface structure was designed to keep the polishing effort as low as possible. The palatal surfaces were kept smooth to ensure oral hygiene

**Figure 9:**
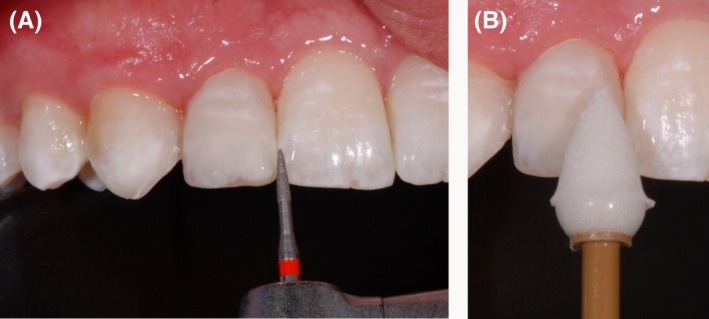
For finishing, only diamond instruments were used (A). The DeTrey^®^ Enhance finishing system and PoGo^®^ One Step Diamond Micro‐Polisher (Dentsply Sirona Inc.) were used for polishing (B)

**Figure 10:**
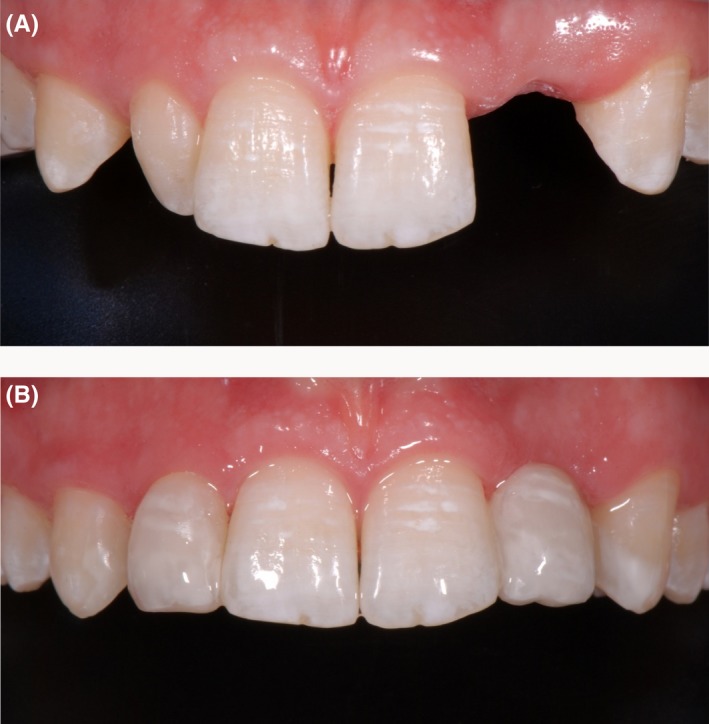
Initial (A) and final situation (B). The left lateral incisor was modeled using a preimpregnated fiberglass ribbon and CeramX^®^ duo. The right lateral incisor and the canines were enlarged with CeramX^®^ duo. The hypomineralizations were imitated and the incisal edges of the lateral incisors were adapted to the middle incisors for a more natural appearance

**Figure 11:**
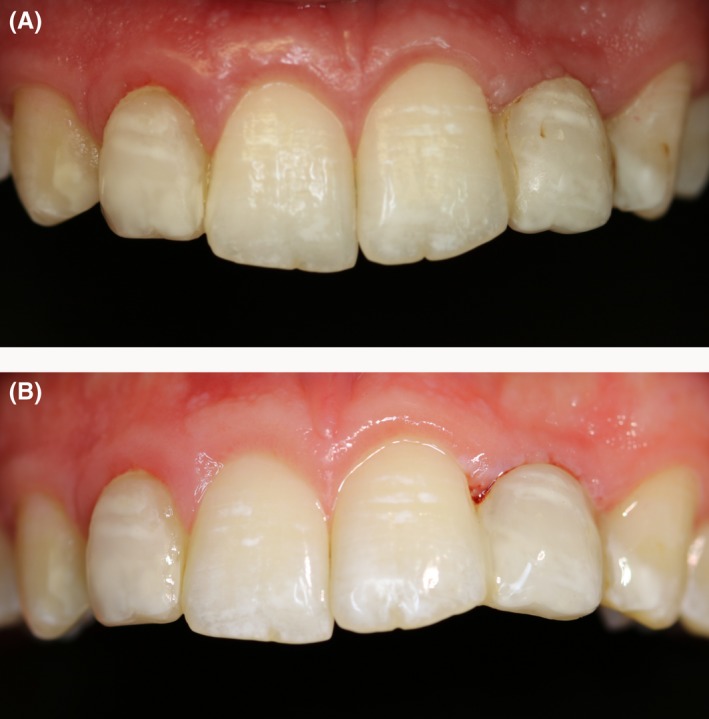
Situation at the follow‐up after 1 year before (A) and after revision (B). Besides some need for revision of the composite restorations, plaque, and gingivitis was diagnosed on the lateral incisors and canines

After finishing the bridgework replacing the left lateral upper incisor, the treatment was continued by remodeling the conically shaped right lateral upper incisor and both upper canines (Figures 6, 7, 8). Occlusion was checked before finishing and polishing. For finishing, only rotating diamond burs were used. The use of polishing disks was avoided in order to achieve a natural surface structure. The DeTrey^®^ Enhance finishing system and PoGo^®^ One Step Diamond Micro‐Polisher (Dentsply Sirona Inc.) were used for polishing (Figure 9A,B). Figure 10A,B show the result of the whole restauration in comparison to the initial situation. After completing the treatment, the patient travelled to Australia for a 1‐year student exchange. At the follow‐up examination after 1 year and immediately after the homecoming of the patient, all restorations were still in service, but needed some revision. Figure 11A shows the situation before and Figure 11B after revision.

## 3 DISCUSSION

Different therapeutic options can be considered for the replacement of missing permanent incisors in young children and adolescents. Implants are often the treatment of choice and should be considered when general and local conditions are favorable.^3^, ^4^ However, implant insertion is generally not intended before the end of the growth period around the age of 18 years.^5^, ^6^ However, dento‐alveolar growth is not strictly depending on chronological age, but occurs parallel to hormonal changes and therefore differs significantly between males and females. Another limiting factor might be the patient's financial background, as dental implants are expensive.^7^ More economically acceptable treatments should therefore be regarded for the replacement of a missing tooth, as a definitive solution or as a long‐term provisional treatment before implant therapy. Removable partial dentures are often considered for very young patients when adjacent teeth are not in their final vertical and horizontal positions. They are not comfortable, however, and according to our experience, are frequently subjected to fracture. When an orthodontic treatment is indicated, an artificial plastic tooth could be attached to a removable or fixed orthodontic appliance to address the aesthetic concern.

The replacement of a missing tooth can also be realized via a conventional bridge or a resin‐bonded fixed partial denture (porcelain fused to metal^8^ or full ceramic^9^).

Fiber‐reinforced composite bridges represent an interesting alternative to conventional metal‐based bridges. They can be realized directly^10^, ^11^, ^12^, ^13^ or indirectly.^14^ In the direct method, an artificial plastic tooth or the avulsed or extracted tooth can be used. An alternative is a direct tooth built‐up using composite resin. The decision to replace the left lateral incisor directly using composite and to fix it unilaterally with a preimpregnated fiberglass ribbon on the left middle incisor was taken together with the patient.

The decision for the unilateral anchorage was based on studies of the group of Kern showing the stability and longevity of single‐retainer anterior bridgework from ceramic.^15^, ^16^, ^17^ The reason to use a single‐retainer construction is that two‐retainer adhesive bridges often fracture at one connector a short time after insertion because of the restricted mobility. This in mind, a single‐retainer construction was also preferred in the present treatment.

An anterior fiber‐reinforced ribbon composite resin bridge was applied to replace the left upper lateral incisor. Furthermore, to get a symmetrical result, the right lateral incisor and the canines were enlarged with composite. This treatment modality presents a quick and efficient alternative method to provide a satisfactory aesthetic appearance with no loss of dental hard tissue. No anesthesia was needed.

The so‐called “Maryland Bridge” has undergone many alterations since its introduction in the 1980s, although the basic advantage of conserving tooth structure has remained. Retention can be improved with a more retentive framework design, the addition of grooves, labial wrap, and the concept of maximum coverage of the enamel. If an implant supported replacement of a single missing incisor is not possible, the Maryland Bridge can still be a restoration of first choice. The main disadvantage of a Maryland Bridge is that it is extremely technique sensitive. Each and every step requires proper planning and precision including impressions and bonding.

To replace a congenitally or traumatically missing permanent anterior tooth, different therapeutic options are available. Fixed anterior fiber‐reinforced composite resin bridges represent one of these options, with many advantages including bondability, reparability, ease of fabrication, and relative longevity. It is considered a minimally invasive procedure with very little loss of dental hard tissue. Compared to traditional prosthetic options, a fiber‐reinforced composite bridge is generally less costly and less technology intensive.

Compared to the metal‐framed Maryland Bridge, a fixed anterior fiber‐reinforced composite resin bridge is easier to bond, more esthetically appealing with no metal shadow, and does not impair the very translucent dental hard tissues in young permanent teeth. A close collaboration with the orthodontist can provide the best local conditions (occlusal relationship) to ensure a long‐lasting result. The use of different dentin and enamel imitating composites to build up the intermediate tooth according to the anatomical layering technique provides a final aspect with natural opalescence, translucency, and opacity. The use of a denture tooth may also be considered instead of direct remodeling the missing tooth. This method is easier, faster and in some cases esthetically more acceptable than the direct fabrication of a tooth. However, in this case the challenge was to imitate the hypomineralizations which would not have been possible with a denture tooth. Furthermore, the shape and the incisal color of denture teeth are difficult to match to the adjacent teeth in some cases.

At the follow‐up after 1 year, the restoration was still in place, but some revisions had to be done. This included some esthetical improvements of the margins of the composite restorations which were detectable and showed some staining. On the palatal side, the fiberglass ribbon was partly exposed and needed to be recovered with composite. In addition, some changes in the natural teeth were obvious at the follow‐up. In comparison to the upper right central incisor, the left one sowed some intrusion and distal tipping, which also included the pontic. It might be speculated that this was the consequence of the unilateral force effect on the incisor caused by the pontic. When comparing the Figure 10 (immediately after restoration) and 11 (1‐year follow‐up), it can be seen that both upper canines showed some abrasion on the tip. However, this was more pronounced for the left canine. As canine guidance was found on both sides, this observation might be the result of a preferred chewing of the left side.

## 4 CONCLUSIONS

In conclusion, the fixed anterior fiber‐reinforced composite resin bridge fabrication technique suggests an alternative treatment option for the temporarily replacement of a missing anterior tooth. Using this technique, it is possible to restore esthetics and function. It is more comfortable than a removable appliance, nonirritating, and hygienic. Generally, it does not require any tooth substance loss and can be repaired, modified, or removed from teeth without any iatrogenic damage. Clinical long‐time studies have to show whether it can also serve as a permanent restoration.

## CONFLICT OF INTEREST

On behalf of all authors, the corresponding author states that there is no conflict of interest.

## AUTHORSHIP

VP: was treating the patient and was preparing the manuscript. SZ: is corresponding author, was preparing the manuscript, performed follow‐up‐examinations, and made revisions. LM: was supervising VP and proofreading the manuscript.
